# Proton NMR visible mobile lipid signals in sensitive and multidrug-resistant K562 cells are modulated by rafts

**DOI:** 10.1186/1475-2867-5-2

**Published:** 2005-02-09

**Authors:** Aurélie Mannechez, Paiboon Reungpatthanaphong, Jacques D de Certaines, Geneviève Leray, Laurence Le Moyec

**Affiliations:** 1 Laboratoire de Résonance Magnétique en Biologie et Médecine, Université Rennes 1, avenue du Professeur Léon Bernard, 35043 RENNES Cedex. France; 2 Laboratoire LPBC-CSSB, UMR CNRS 7033, Université Paris 13, 74 avenue, Marcel Cachin, 93017 Bobigny Cedex. France

**Keywords:** Proton Magnetic Resonance Spectroscopy, Multidrug resistance, Mobile lipids, Rafts

## Abstract

**Background:**

Most cancer cells are characterized by mobile lipids visible on proton NMR (^1^H-NMR), these being comprised mainly of methyl and methylene signals from lipid acyl chains. Erythroleukemia K562 cells show narrow signals at 1.3 and 0.9 ppm, corresponding to mobile lipids (methylene and methyl, respectively), which are reduced when K562 cells are multidrug resistant (MDR). While the significance of the mobile lipids is unknown, their subcellular localization is still a matter of debate and may lie in the membrane or the cytoplasm. In this study, we investigate the role of cholesterol in the generation of mobile lipid signals.

**Results:**

The proportion of esterified cholesterol was found to be higher in K562-sensitive cells than in resistant cells, while the total cholesterol content was identical in both cell lines. Cholesterol extraction in the K562 wild type (K562wt) cell line and its MDR counterpart (K562adr), using methyl-β-cyclodextrin, was accompanied by a rise of mobile lipids in K562wt cells only. The absence of caveolae was checked by searching for the caveolin-1 protein in K562wt and K562adr cells. However, cholesterol was enriched in another membrane microdomain designated as "detergent-insoluble glycosphingomyelin complexes" or rafts. These microdomains were studied after extraction with triton X-100, a mild non-ionic detergent, revealing mobile lipid signals preserved only in the K562wt spectra. Moreover, following perturbation/disruption of these microdomains using sphingomyelinase, mobile lipids increased only in K562wt cells.

**Conclusion:**

These results suggest that cholesterol and sphingomyelin are involved in mobile lipid generation via microdomains of detergent-insoluble glycosphingomyelin complexes such as rafts. Increasing our knowledge of membrane microdomains in sensitive and resistant cell lines may open up new possibilities in resistance reversion.

## Background

When studied by NMR proton spectroscopy, most cancer cells are characterized by increased narrow signals at 0.9 and 1.3 ppm corresponding, respectively, to methyl and methylene resonances that belong to lipid acyl chains moving isotropically. This so-called "mobile lipid signal" (or ML signal) has been studied for several decades (for review see[1]). However, research has failed to elucidate the molecular origin of mobile lipids, their subcellular localization or their physiological significance.

Regarding their molecular origin, acyl chains can form part of triglycerides or esterified cholesterol. Phospholipidic acyl chains may also be involved if they are not embedded in membrane lipid bilayers. As far as localization is concerned, two types of subcellular origin are still debated. As early as 1988 [2], Mountford et al proposed an origin in lipoprotein-like microdomains within the plasma membrane. More recently, some authors [3] have suggested the occurrence of cytosolic droplets, which appear concomitantly with the ML in stressed cells [4] or which are associated with necrosis [5] and apoptosis. However, it has been shown that ML intensity is not always correlated with the number of cytoplasmic droplets [6]. These studies (op cit.) tend to show that cytosolic lipid droplets generate mobile lipid signals in NMR spectra, without excluding the possibility that such signals could arise independently of the presence of cytosolic lipid bodies.

^1^H-NMR spectroscopy shows that ML are decreased in erythroleukemia K562 cells when these become resistant (K562adr)[7], whereas the compositions of lipids extracted from K562wt (drug sensitive) and K562adr cells are the same, except for sphingomyelin content which is increased in resistant cells [8]. Thus, although we may explain this by a structural difference in lipid "organization", the significance of such a behaviour is not yet elucidated. In a previous study [9], we showed that the ML signal is not linked to the externalization of phosphatidylserine (a membrane phospholipid), this being a phenomena that precedes apoptosis.

In model membranes, free cholesterol interacts with phospholipids and sphingolipids to influence membrane fluidity [10]. *In vivo*, cellular-free cholesterol is located in the plasma membrane [11], which exhibits increasing structural order as demonstrated in erythrocytes [12], LM and CHO cells [13,14]. Moreover, cholesterol in model membranes is able to promote microdomains towards an intermediate state called the liquid-ordered phase (Lo), with less fluidity than the gel phase state and more fluidity than the surrounding membrane in the liquid crystalline state [15].

The aim of this study was to investigate a possible contribution from the cholesterol-containing membrane domain to the ML detected in the NMR proton spectra of wild-type K562 and K562 adriamycin-resistant cells.

In a first set of experiments, cell membrane cholesterol was extracted by methyl-β-cyclodextrin (MCD). MCD is an oligosaccharid able to pump cholesterol out of the cell [16]. Moreover, cholesterol extracted by MCD originates primarily from the plasma membrane [17]. Cell incubation with MCD was expected to change the membrane microdomains via cholesterol extraction, and consequently produce variations in ML. In a second set of experiments, we used triton-X100 to separate membrane microdomains and then investigated their role in ML generation. In this study, we use the term "Detergent Insoluble Glycosphingolipid Complexes" (DIGCs) or "rafts" to refer to the membrane microdomains isolated by treatment with this mild non-ionic detergent (for review see [18]). Finally, as these raft microdomains are also enriched in sphingolipids, we used sphingomyelinase to modify their "organization", with the aim of inducing changes in ML signal intensity.

## Results

### Cholesterol quantification

K562wt and K562adr cells do not differ in their total cholesterol contents. The proportion of cholesterol-ester were found to be lower in the resistant cells than in the wild type cells (Table 1). This implies that K562wt cells contain more free cholesterol than K562adr cells. Total cholesterol content after MCD treatment (Table 1) shows a 45 % decrease in both K562wt and K562adr cells with respect to the control.

**Table 1:** Quantification of cholesterol in K562 cells. Results are expressed as means ± SD.

	**K562 wt**	**K562adr**
Total cholesterol (μg/10^6 ^cells)	3.6 ± 0.5 (n = 5)	3.5 ± 0.3 (n = 4)
Cholesterol ester (percentage of total cholesterol)	26.5 ± 1.3 (n = 6)	19.4 ± 2.4 (n = 5)
Cholesterol variation with MCD treatment (% of control)	57 ± 13 (n = 5)	54 ± 15 (n = 4)

### Cell proliferation after incubation with methyl-β-cyclodextrin

**Figure 1:**
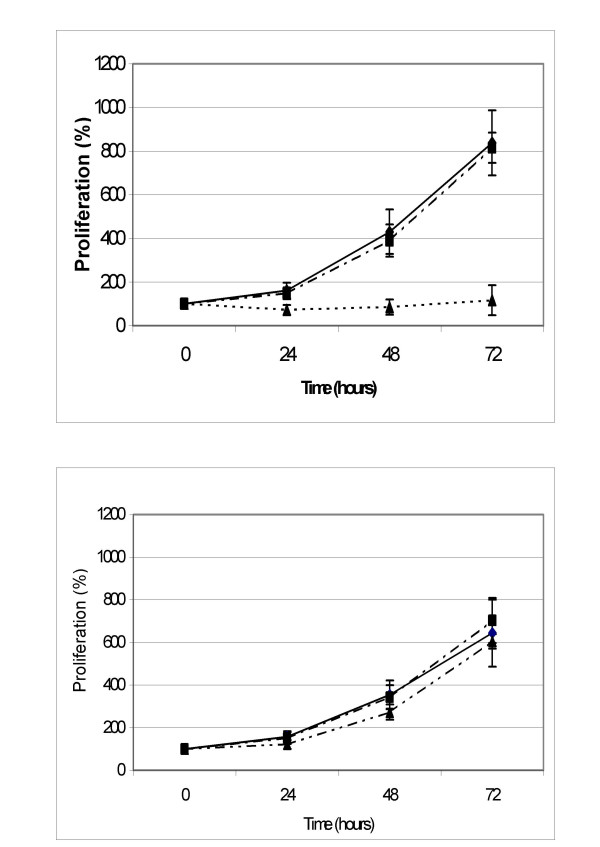
K562wt cells (up) and K562adr cells (down) were incubated for 2 hours in medium alone (WT RPMI; full line and square, n = 6) supplemented in FCS 10% (dot line and lozenge, WT R10; n = 6), in methyl-β-cyclodextrin 5 mM (dot line and triangle, WT MCD; n = 6) and successively incubated in R10 for 72 hours. Data points are the percentages of the cellular concentration normalized to cellular concentration at T = 0 hours expressed as means, with vertical bars representing standard deviation (SD).

**Figure 2:**
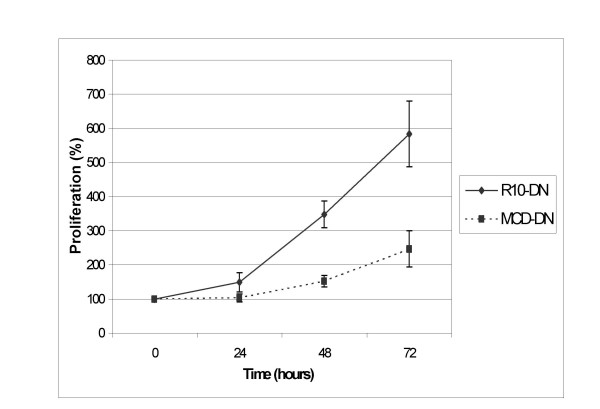
K562adr resistance modulation after MCD treatment. K562adr cells were incubated for 2 hours in medium supplemented in SVF 10% (R10-DN; n = 4) or in methyl-β-cyclodextrin 5 mM (CD-DN; n = 4) and seeded in R10 containing daunorubicin for 72 hours. Data points are the percentages of the cellular concentration normalized to cellular concentration at T = 0 hours expressed as means, with vertical bars representing standard deviation (SD).

The cell proliferation results for K562wt and K562adr are presented in Figs. 1 and 2. The two-hour incubation without FCS had no effect on proliferation in K562wt or K562adr when compared to cells incubated in RPMI+FCS. Treatment with MCD inhibited proliferation in K562wt. This was not the case with K562adr, which grew normally.

When K562adr cells were resuspended in R10 containing daunorubicin (0.25 nM), they were able to proliferate at this daunorubicin concentration. However, after treatment with MCD, the K562adr proliferation rate was decreased as shown in figure 2. This shows that MCD treatment reversed the resistance phenotype in K562adr; despite having no effect on cell growth, MCD affected the K562adr cell membrane.

### Caveolin-1 expression: Western-Blot

Caveolin-1 expression was studied in K562 cells by SDS-page and western-blotting. Caveolin-1 is a protein of 21 kD [19]. As a positive control, we chose a protein extract of NIH-3T3 cells that are known to express caveolin-1. In this control, a band appeared between the 19 and 32 kD size markers. In the present study, however, neither K562wt nor K562adr expressed caveolin-1.

### ^1^H-NMR spectra

#### Incubation with methyl-β-cyclodextrin

**Figure 3:**
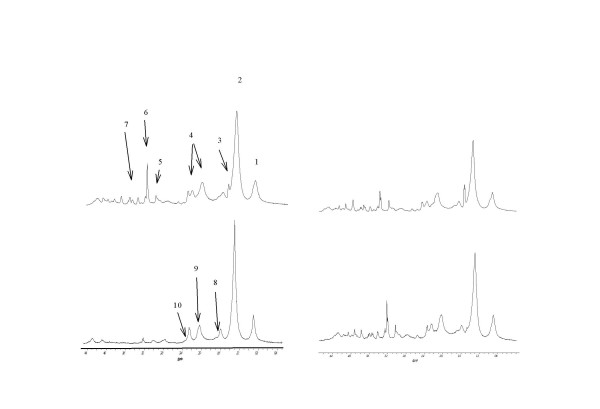
NMR proton spectra obtained at D3 on K562wt (left side) and K562adr (right side) on control cells (top spectra) or treated with 5 mM MCD during 2 hours (bottom spectra). Peak assignment – 1: CH3 from fatty acid chains; 2: CH2 from fatty acid chains; 3: Alanine; 4 : glutamine and glutamate; 5: creatine; 6 N-trimethyl from choline, 7: Inositol; 8 : CH_2 _in β position from ester function in fatty acid ester; 9: CH2 in a position of a double bond in fatty acids; 10 : CH_2 _in α position from ester function in fatty acid ester.

Figure 3 shows spectra obtained on K562wt and K562adr cells, and resonance ratios are presented in Table 2. These results show that MCD increases the lipid signals only in K562wt cells. This increase can also be seen in Table 3, which reports a significant increase of CH_3_/Ct and CH_2_/Ct ratios in MCD-treated K562wt cells compared with non-treated cells.

**Table 2:** NMR peak ratios measured on K562wt and K562adr spectra. Cells were incubated for 2 hours in RPMI, or in RPMI containing methyl-β-cyclodextrin 5 mM (MCD). Results are expressed as means of arbitrary units ± SD multiplied by tenStatistical analysis : *: P < 0.05 when compared with RMPI incubated cells.

	**K562wt**		**K562adr**	
**Group**	RPMI *(n = 6)*	MCD *(n = 5)*	RPMI *(n = 4)*	MCD *(n = 4)*

CH_3_/Ct	50 ± 5	101 ± 22*	34 ± 3	34 ± 2
CH_2_/Ct	247 ± 35	537 ± 13*	142 ± 22	130 ± 18
N^+^(CH_3_)_3_/Ct	23 ± 3	22 ± 7	17 ± 3	21 ± 16

**Table 3:** NMR peak ratios measured on K562wt and K562adr spectra. PBS: incubation in PBS; PFA: incubation in PFA; Triton : incubation in PFA and triton X; Smase: incubation in PFA, triton X-100 and 0.5 units sphingomyelinase. Results are expressed as means of arbitrary units ± SD multiplied by ten.

	**K562wt**				**K562adr**			
**Group**	**PBS ***(n = 4)*	**PFA ***(n = 7)*	**Triton ***(n = 7)*	**Smase ***(n = 4)*	**PBS ***(n = 4)*	**PFA ***(n = 7)*	**Triton ***(n = 8)*	**Smase ***(n = 5)*

CH_3_/Ct	198 ± 21	231 ± 12	312 ± 42*	392 ± 105°	161 ± 19	192 ± 22	265 ± 62*	267 ± 42*
CH_2_/Ct	541 ± 52	611 ± 78	1060 ± 164*	1696 ± 441°	308 ± 93	386 ± 56	695 ± 141*	565 ± 167*
N(CH_3_)_3_/Ct	132 ± 6	141 ± 18	53 ± 10*	79 ± 17°	114 ± 12	132 ± 36	60 ± 15*	94 ± 39*

Statistical analysis : * : P < 0.05 when compared with PBS-incubated cells,

°: P < 0.05 when compared with Triton-treated cells,

#### Triton X-100 and sphingomyelinase treatments

**Figure 4:**
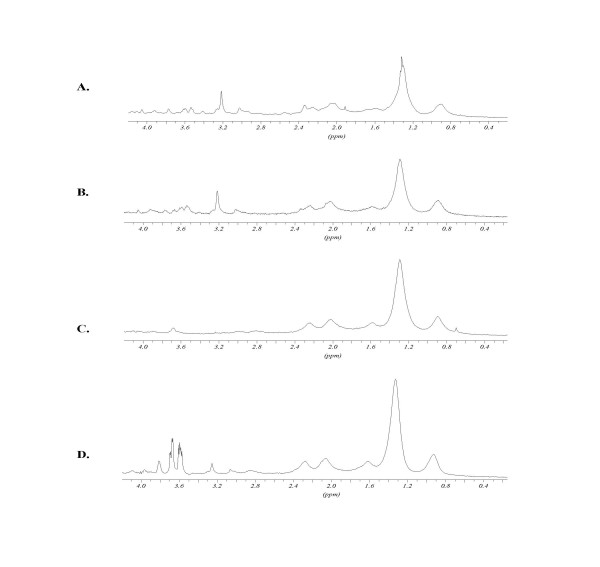
K562wt ^1^H-NMR spectra: effects of Triton X-100 and sphingomyelinase treatments. A: non-treated cells; B: cells fixed with PFA; C: cells fixed with PFA 4% in triton-X100 1%; D: cells fixed with PFA 4% incubated in triton-X100 1% and with 0.5 units sphingomyelinase. For peak assignment, see figure 4. Peaks at 3.6 ppm after SMase treatment arise from enzyme working buffer.

**Figure 5:**
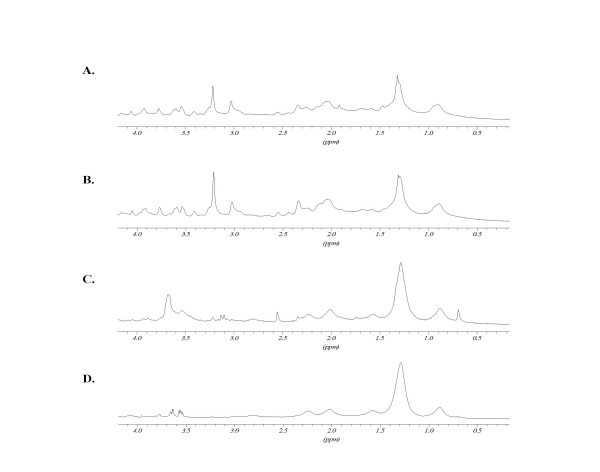
K562adr ^1^H-NMR spectra: effects of Triton X-100 and sphingomyelinase treatments. A: non-treated cells; B: cells fixed with PFA; C: cells fixed with PFA 4% in triton-X100 1%; D: cells fixed with PFA 4% incubated in triton-X100 1% and with 0.5 units sphingomyelinase. H-NMR spectra: Triton X-100 and sphingomyelinase treatment. For peak assignment, see Fig. 4. Peaks at 3.6 ppm after SMase treatment arise from enzyme working buffer.

spectra obtained after treatments with PFA, triton and sphingomyelinase are presented in Figures 4 and 5 for K562wt and K562adr cells, respectively. The ratios of the peak intensities with respect to the creatine peak are reported in Table 3. When compared to the control, PFA treatment did not modify the cell spectra. After microdomain separation by triton treatment, the CH_2_/Ct ratio was increased for K562wt cells and, to a lesser extent, for K562adr cells. N-trimethyl to creatine peak ratios were decreased in both cell lines. After treatment of these microdomains with sphingomyelinase, the spectra showed an increased CH_2_/Ct ratio compared with the previous spectrum obtained on "DIGCs" in K562wt cells. However, the spectra of K562adr cells treated with Smase remained unchanged compared with spectra obtained on DIGCs from K562adr cells.

## Discussion

We studied the modification of cell spectra using cholesterol and sphingomyelin because: i) cholesterol levels affect membrane fluidity; ii) membrane microdomains, rafts and caveolae are all enriched in these lipids. Western-blot did not detect the presence of caveolin-1 in either K562wt or K562adr. This confirms previous results showing the absence of caveolin-1 in K562wt [20]. Even if membrane microdomains exist in this cellular type, this implies they represent rafts and not caveolae.

Rafts have been described as corresponding to membrane microdomains enriched in cholesterol and sphingomyelin lipids [18]. In our first set of experiments, we disrupted the rafts using MCD to extract cholesterol from the cells. Before MCD treatment, K562wt and K562adr cells displayed the same cholesterol concentrations, which were found to be in agreement with previous results obtained for other cancer cells [21,22]. In K562wt cells, this cholesterol was preferentially in the form of cholesterol-ester, implying that the absolute concentration of free cholesterol is lower in K562wt cells compared with K562adr cells. 90% of the free cholesterol is contained in plasma membrane [11]. On the other hand, cholesterol-ester synthesized in the endoplasmic reticulum is generally localized in the cytosol and is able to form cytoplasmic lipid bodies. This cholesterol is less available to MCD extraction. Thus, MCD treatment may affect the free-cholesterol plasma membrane pool [17] and not the intracellular pool that mainly contains cholesterol-ester in K562wt cells. Consequently, in our study, a higher proportion of membrane cholesterol (corresponding to free cholesterol) was extracted in K562wt cells compared against K562adr cells. This may account for the fact that the proliferation rate for K562wt was more affected than for K562adr. Moreover, the removal of a greater proportion of membrane cholesterol modified the NMR proton spectra of the cells, since the mobile lipid signals only increased in the K562wt cells.

Nevertheless, we observe that MCD treatment affected the K562adr cell line. This is demonstrated by the reversion of daunorubicin resistance. In the plasma membrane, K562adr cells express the P-gp glycoprotein responsible for the extrusion of drugs. When K562adr cells were treated with MCD, the resistance to daunorubicin was decreased, showing that the P-gp is unable to extrude daunorubicin from the cells. P-gp has been widely reported to be sensitive to the lipid content of plasma membrane [23]. The present study shows that MDR is sensitive to the cellular cholesterol content. Indeed, it has been shown that drugs can accumulate in MDR cells grown in the presence of MCD [24].

This first set of experiments suggests that ML signals are generated during the disruption of membrane microdomains in K562wt cells, because MCD has been shown to extract cholesterol preferentially from membrane microdomains [17]. Overall, these results tend to show a link might exist between microdomains and the ML signal in K562 cells.

In a second set of experiments, we isolated rafts using triton X-100 at 4°C, a mild detergent in which they are insoluble [25]. We applied this method to K562wt and K562adr cells. NMR spectra obtained on these isolated raft fractions show an increase of the ML signals for K562wt and K562adr cells. In this particular case, this implies that ML could be produced by raft domains. Although it has been shown that raft structures are tightly packed at 4°C, there is no report of their ?organization/structure at 25°C, which is the temperature chosen for the NMR experiments [26]. At this temperature, microdomain lipids could be in another physical state allowing them to move more isotropically and produce an NMR-visible signal: their solubility in triton at 25°C supports this hypothesis [27].

In the final set of experiments, we incubated detergent insoluble fractions with sphingomyelinase, which was supposed to metabolise the sphingomyelin included in the rafts. Only the K562wt spectra exhibited an increase in ML signal compared with the raft spectra. Again, this suggests that microdomains are differently organized between K562wt and K562adr. Several hypotheses may be proposed to explain the difference between K562wt and K562adr: i) sphingomyelin concentrations are higher in K562adr; ii) lipid microdomains in K562adr may be organized in such a way that sphingomyelin is inaccessible to sphingomyelinase and iii) there are more microdomains in K562adr, as suggested previously by some authors [28].

## Conclusions

These results suggest that, while isolated rafts generate ML signals within the membrane, the presence of rafts seems to maintain a relatively tight organization. When this organized structure is disrupted by MCD or sphingomyelinase, the ML signal may be detected in K562wt cells and the P-gp function can then be modulated in K562adr cells. Taken together, the results suggest that ML signals may be sensitive to modifications in the lipidic organization of the cells and membrane, in which microdomains behave as rafts enriched in cholesterol and sphingomyelin. A better knowledge of these microdomains, as well as the differences in behaviour between sensitive and resistant cells, could open up new therapeutic perspectives for reversing the drug-resistance phenotype.

## Materials and Methods

### Chemicals

Methyl-β-cyclodextrin, sphingomyelinase, cholesterol, chloroform, isopropanol and paraformaldehyde, as well as deuterium oxide (D_2_O) and all chemicals for western-blot analysis were provided by Sigma-Aldrich (Saint-Quentin Fallavier, France). Triton X-100 was supplied by Merck (Schuchardt, Darmstadt, Germany), and methanol by Acros Organics (Geel, Belgium).

### Cell culture

Human erythroleukemia K562 cells were grown in the culture medium RPMI 1640 (Bio-Whittaker Europe, Verviers, Belgium) supplemented with fœtal calf serum (FCS) (10%) and glutamine (2 mM) (R10 medium) at 37°C and 5 % CO_2_. The K562 resistant variant cells (K562adr) were a gift from F. Calvo, Université Paris 7, and were grown in the same medium with 0.5 μg/ml adriamycin.

### Cholesterol quantification

Lipids were extracted as previously described [29], and then desiccated and resuspended in isopropanol. Total cholesterol and free cholesterol were assayed using a kit (Cholesterol RTU, Biomérieux Craponne, and Boeringher, France) based on cholesterol esterase. This was followed by cholesterol oxidase reaction and colour development measured on an absorption spectrometer at 500 nm. Concentration was determined using a standard solution of 0.5 mg/ml cholesterol. We first checked that the solvent (isopropanol or chloroform) did not have any effect on colour development.

### Cholesterol extraction using methyl-β-cyclodextrin

Cells were incubated in RPMI, glutamin 2 mM, containing methyl-β-cyclodextrin 5 mM for 2 hours at 37°C, 5% CO_2_. Controls were incubated in RPMI, glutamin 2 mM, with or without FCS 10% for 2 hours.

### Proliferation

After incubation with methyl-β-cyclodextrin, with RPMI+ FCS or with RPMI (the last being used as a control for RPMI+FCS, considering that methyl-β-cyclodextrin is not used in the medium with FCS), cells were collected, washed and resuspended at 150,000 cells/ml in RPMI, glutamin 2 mM, FCS 10%. Cells were seeded in triplicate on 96-well microplates and incubated at 37°C and 5% CO_2_. Proliferation was assessed by counting cells with a cell coulter (Beckman Coulter, ZI model) at 24, 48 and 72 hours after seeding.

### Caveolin-1 detection: western-blot

Cells were collected in late log-phase and washed twice in PBS 150 mM and were resuspended in loading buffer (urea 4 M, lauryl sulphate 3.8%, glycerol 20%, tris base 75 mM pH 6; β-mercaptoethanol 5%; bromophenol blue 5%) for 5 mn at 95°C. They were centrifuged at 12500 g, 4°C for 10 minutes, and 50 μg of extracted protein were deposited per well on an 8% acrylamide gel. Migration was performed in an electrophoresis buffer (TRIS base 125 mM; glycine 250 mM; lauryl-sulphate 0.1%, pH 8.1) for 1 hour at 125 V with a size marker (Kaleidoscope prestained standards Bio-rad). Protein was transferred on a nitrocellulose membrane for 2 hours at 80 V and 4°C in a transfer buffer (tris-base 48 mM; glycine 39 mM, SDS 0.037%, methanol 20%). Non-specific sites were blocked with 1% milk (Regilait, skimmed milk, Saint-Martin-Belle-Roche, France) in TBS 1X (tris-base 20 mM, NaCl 500 mM, and pH 7.5). The nitrocellulose membrane was incubated for 1 hour at room temperature with 1 μg/ml anti-caveolin 1 rabbit antibody (Tebu, France) and washed three times for 10 minutes with TBS before incubation for 1 hour at room temperature with goat anti-rabbit and conjugate secondary antibody (1:3000). After three washes in TBS for 10 minutes, staining was developed in a phosphatase alkaline buffer (NaHCO_3 _0.1 M; MgCl_2_, 6H_2_O 1 mM) with tetrazolium nitro-blue 3% in N,N-dimethylformamide 70% and bromo-4-chloro-3-indolylphosphate p-toluidine 1% in N,N-dimethylformamide 70% for 30 minutes at room temperature in the dark.

### Triton treatment

Cells were collected at the end of the log-phase, 4 days after seeding, when ML were at a maximum, and washed twice with 1 ml PBS 150 mM at 4°C. They were resuspended in paraformaldehyde (PFA) 3% in PBS 150 mM for 30 minutes at 4°C and washed twice with 1 ml PBS 150 mM at 4°C to be resuspended in triton X-100 1% in PBS 150 mM at 4°C for 30 minutes. Cells or triton-insoluble fractions were collected and washed for NMR experiments at 25°C.

### Sphingomyelinase treatment

After triton treatment, cells were collected, washed with PBS 150 mM and treated for 35 mn at 37°C with 0.5 units of sphingomyelinase. Cells were washed as described for NMR experiments at 25°C.

### NMR analysis

Cells were washed twice in 1 ml PBS 150 mM, twice in PBS/D_2_O, centrifuged at 250 g and counted. Then, 10^7 ^cells were resuspended in 400 μl PBS/D_2_O before transfer to a 5-mm Shigemi NMR tube. Experiments were performed without rotation, and the ?analysed cell pellet was maintained in the coil volume in the Shigemi NMR tube.

The NMR proton spectra of whole cells were obtained at 25°C on a Unity Inova spectrometer (Varian, France) working at 500 MHz. One-dimensional runs were performed by accumulating 128 transients of 90° pulse with 2 s relaxation time. The signal from the residual water was suppressed by the presaturation technique, by using 0.03 mW irradiation for 2 s. Acquisition time was 1.34 s on 16K data points, corresponding to a spectral width of 6 kHz. The Fourier transform was applied without zero-filling using an exponential window multiplication function corresponding to 1 Hz line broadening. The resonances were integrated after automatic baseline correction. Two-dimensional COSY runs were performed with 2K data points in the F2 direction and 256 data points in the F1 direction. The two-dimensional Fourier transformation was applied after zero filling to 512 data points in the F1 direction with a sine-bell function in both directions. Each run consisted of a one-dimensional acquisition and a two-dimensional COSY spectrum. Peak assignments were based on data from the literature. The peak areas were measured by manual integration with the Bruker WINNMR software using a manual tangential baseline correction for each peak, and the assigned peak areas were normalized to the creatine peak area. The following resonances were integrated: methyl group (CH_3 _at 0.9 ppm), methylene group (CH_2 _at 1.3 ppm), choline N-trimethyl group (N^+^(CH_3_)_3 _at 3.2 ppm) and creatine (CH_3 _at 3.05 ppm). The values obtained for the different treatments of the two cells lines were compared by ANOVA analysis followed by a Student-Neumann-Keuls test for group-to-group comparison. P < 0.05 was considered as a significant value.

NMR proton spectra of PFA or triton- or SMase-treated cells were acquired at 500 MHz and 25°C on a Bruker Advance DMX500 spectrometer (Bruker, Wissembourg, France). The runs consisted of a 1D acquisition with similar parameters as the previous 1D spectra, and the spectrum analysis was performed as previously with Bruker WINNMR software.

The possible contribution of lactate to the 1.3 ppm signal was eliminated by analysing 2D Cosy spectra in whole cells, which resolved the resonances of lactate from fatty acid chains. In fixed cells, we calculated the ratio of the double-bond signal (CH = CH at 5.4 ppm) to CH_2 _peak area. As both groups belong to fatty acyl chains, this ratio remained constant since lactate did not significantly contribute to the increase of the CH_2 _signal [1].

## List of abbreviations

NMR: Nuclear Magnetic Resonance; MDR: multi-drug resistance; ML: mobile lipid; K562wt: K562 wild type; K562adr: K562 adriamycin-resistant; MCD: methyl-β-cyclodextrin; DIGC: detergent-insoluble glycosphingolipid complex; FCS: fœtal calf serum; R10: RPMI culture medium with 10% FC; PBS: Phosphate buffer saline; TRIS: Tris(hydroxymethyl)-1,3-propanediol; TBS: TRIS buffer Saline; PFA: paraformaldehyde; COSY: correlated-spectroscopy; CH_3_: methyl; CH_2_: methylene; N(CH_3_)_3_: N-trimethyl; Ct: creatine

## Authors' contributions

all authors contributed equally to this work
